# Characterization of BRCA1/2-Directed ceRNA Network Identifies a Novel Three-lncRNA Signature to Predict Prognosis and Chemo-Response in Ovarian Cancer Patients With Wild-Type BRCA1/2

**DOI:** 10.3389/fcell.2020.00680

**Published:** 2020-07-29

**Authors:** Meiling Zhang, Guangyou Wang, Yuanyuan Zhu, Di Wu

**Affiliations:** ^1^Department of Obstetrics and Gynecology, The First Affiliated Hospital of Harbin Medical University, Harbin, China; ^2^Department of Neurobiology, Heilongjiang Provincial Key Laboratory of Neurobiology, Harbin Medical University, Harbin, China; ^3^Department of Pathology, Harbin Medical University, Harbin, China

**Keywords:** ovarian cancers, long non-coding RNAs, BRCA1/2, ceRNA, signature

## Abstract

Long non-coding RNAs (lncRNAs) have been reported to interact with BRCA1/2 to regulate homologous recombination (HR) by diverse mechanisms in ovarian cancers (OvCa). However, genome-wide screening of BRCA1/2-related lncRNAs and their clinical significance is still unexplored. In this study, we constructed a global BRCA1/2-directed lncRNA-associated ceRNA network by integrating paired lncRNA expression profiles, miRNA expression profiles, and BRCA1/2 expression profiles in BRCA1/2 wild-type patients and identified 111 BRCA1/2-related lncRNAs. Using the stepwise regression and Cox regression analysis, we developed a BRCA1/2-directed lncRNA signature (BRCALncSig), composing of three lncRNAs (LINC01619, DLX6-AS1, and AC004943.2) from the list of 111 BRCA1/2-related lncRNAs, which was an independent prognostic factor and was able to classify the patients into high- and low-risk groups with significantly different survival in the training dataset (HR = 2.73, 95 CI 1.65–4.51, *p* < 0.001). The prognostic performance of the BRCALncSig was further validated in the testing dataset (HR = 1.9, 95 CI 1.21–2.99, *p* = 0.005) and entire TCGA dataset (HR = 2.17, 95 CI 1.56–3.01, *p* < 0.001). Furthermore, the BRCALncSig is associated with chemo-response and was also capable of discriminating nonequivalent outcomes for patients achieving complete response (CR) (log-rank *p* = 0.003). Functional analyses suggested that mRNAs co-expressed with the BRCALncSig were enriched in cancer-related or cell proliferation-related biological processes and pathways. In summary, our study highlighted the clinical implication of BRCA1/2-directed lncRNAs in the prognosis and treatment response of BRCA1/2 wild-type patients.

## Introduction

Ovarian cancer (OvCa), one of the most common cancers in the genital system, is the fifth leading cause of cancer death in females. It is estimated that there are 22,530 newly diagnosed cases, and 13,980 deaths occurred in the United States according to the cancer statistics 2019 (Siegel et al., 2019). The standard treatment of advanced OvCa is surgical tumor debulking, followed by platinum/taxane-based chemotherapy. However, most patients after therapy will suffer disease relapse and face a poor outcome with the overall 5-year survival rate of approximately 30% due to the lower complete response (CR) rate of 40–60% to first-line chemotherapy (Cancer Genome Atlas Research and Network, 2011; Kim et al., 2012; Sun J. et al., 2019). Therefore, identifying potential molecular biomarkers to predict patients’ outcomes and chemotherapy response will be critical for guiding individualized treatment of patients with poor outcomes and drug resistance.

BRCA1 and BRCA2 (abbreviated as BRCA1/2) are well-known tumor suppressor genes and play essential roles in DNA repair and damage response mainly through homologous recombination (HR) (Powell and Kachnic, 2003; Yoshida and Miki, 2004). Therefore, BRCA1/2 mutation is associated with HR deficiency and genomic instability, leading to favorable outcomes for OvCa patients harboring BRCA1/2 mutation when subjected to platinum-based treatment compared with BRCA1/2 wild-type patients (McCabe et al., 2006; Lu et al., 2014; da Cunha Colombo Bonadio et al., 2018). However, BRCA1/2 are essential for normal tissue cells, and therefore, even though cancer patients without BRCA mutation possibly have a better outcome after platinum-based treatment compared with those with BRCA mutation. Long non-coding RNAs (lncRNAs) is a newly discovered class of non-coding RNAs (ncRNAs) and play crucial roles in a wide range of biological processes by fulfilling regulatory roles at the transcriptional, post-transcriptional or epigenetic levels (Kornienko et al., 2013; Mercer and Mattick, 2013). A large number of studies have suggested that the aberrant expression of lncRNAs contribute to the development and progression of human cancers, and can serve as novel biomarkers in cancer diagnosis, prognosis and treatment response (Zhou et al., 2015a,b, 2016a,b, 2017, 2018a,b,c). Recent studies have reported that lncRNAs can interact with BRCA1/2 to regulate HR by diverse mechanisms (Sharma et al., 2015; Wu and Wang, 2017; Su et al., 2018; Bao et al., 2019). For example, Sharma et al. (2015) found that lncRNA (DNA damage-sensitive RNA1) can regulate HR and DNA damage response by interacting with BRCA1 and *hnRNPUL1*. Another lncRNA *PCAT-1* was reported to have a functional deficiency in HR through its repression of the BRCA2 (Prensner et al., 2014). It is well known that lncRNAs can act as competing for endogenous RNA (ceRNA) to communicate with mRNAs by competing for binding to shared microRNAs (miRNAs) (Liu et al., 2018). However, BRCA1/2-directed lncRNA-associated ceRNA network and their implication in prognosis and treatment response of BRCA1/2 wild-type patients remain mostly unknown.

In this study, we tried to construct and analyze a global BRCA1/2-directed lncRNA-associated ceRNA network by integrating paired lncRNA expression profiles, miRNA expression profiles, and BRCA1/2 expression profiles in BRCA1/2 wild-type patients, and identified potential BRCA1/2-directed lncRNA biomarkers for predicting prognosis and chemotherapy response of BRCA1/2 wild-type patients.

## Materials and Methods

### OV Patient Datasets

A total of 459 OvCa patients and their clinical information, miRNA expression profiles, lncRNA expression profiles, mRNA expression profiles, somatic mutation and copy number variation information of *BRCA1* and *BRCA2* were downloaded from UCSC Xena^1^. After examining BRCA1/2 mutation information, these OvCa patients included 217 patients with BRCA1/2 mutation and 242 patients with wild-type BRCA1/2, which were randomly divided into a training dataset (*n* = 121) and a testing dataset (*n* = 121).

### Construction of BRCA1/2-Directed ceRNA Network

The high-quality experimentally validated miRNA-BRCA1/2 target association data was obtained from Tarbase (version 8.0)^2^ (Karagkouni et al., 2018), miRTarBase (version 8.0)^3^ (Huang et al., 2020) databases. The high-quality experimentally validated miRNA-lncRNA target association data was obtained from ENCORI database^4^ (Li et al., 2014). The BRCA1/2-directed ceRNA network was built according to the “ceRNA hypothesis” as follows: (?) Pearson correlation coefficient (PCC) was calculated between BRCA1/2 and each lncRNA, and those BRCA1/2-lncRNA pairs with positive correlation and *P*-value < 0.05 was selected for further analysis; (?) A BRCA1/2-lncRNA pair in which both BRCA1/2 and lncRNA are regulated by more than one same miRNAs was selected as candidate BRCA1/2-lncRNA ceRNA pairs; (?) If both BRCA1/2 and lncRNA in this candidate BRCA1/2-lncRNA ceRNA pair were co-expressed negatively with a same miRNAs, this BRCA1/2-miRNA-lncRNA was defined as BRCA1/2-directed ceRNA triples; (?)These BRCA1/2-directed ceRNA triples was integrated to form a global BRCA1/2-directed ceRNA network (BRCA-CeNet).

### Development of a BRCA1/2-Directed lncRNA Signature

The univariate Cox regression analysis was used to evaluate the association between lncRNA in a BRCA1/2-directed ceRNA network and survival. Then a stepwise regression was used to identify an optimal lncRNAs combination according to Akaike information criterion (AIC) as prognostic lncRNAs biomarkers. Finally, optimal prognostic lncRNAs were fitted in a multivariate Cox regression analysis to evaluate their relative contribution to survival prediction. Finally, a BRCA1/2-directed lncRNA signature (BRCALncSig) was developed by the linear combination of the expression value of optimal prognostic lncRNAs with multivariate Cox regression coefficient as the weight (Sun et al., 2020). The BRCALncSig classified patient into the high-risk group and low-risk group using the median value of the training dataset as risk cutoff.

### Statistical Analysis

Kaplan–Meier survival curves and the log-rank test were used to compare survival differences between predicted high-risk group and low-risk group using the R package “survival.” Univariate and multivariate analyses were performed using Cox proportional hazards regression model. Chi-squared tests are used to test for differences between two groups. Functional enrichment analysis for GO and KEGG was performed using the R package “clusterProfiler” (Yu et al., 2012).

## Results

### Construction and Analysis of BRCA1/2-Directed ceRNA Network

**FIGURE 1:**
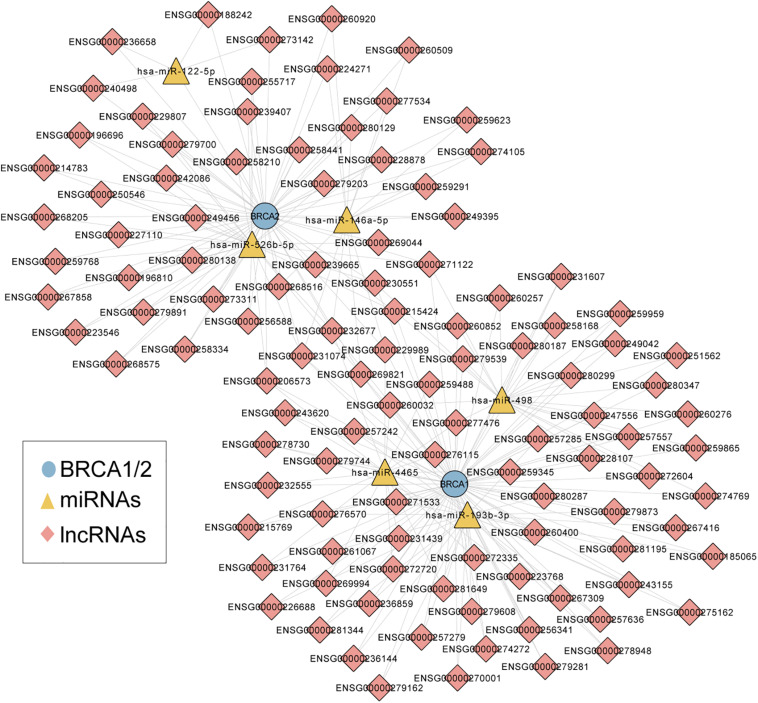
A global view of BRCA1/2-directed ceRNA networks comprised of 119 nodes (including BRCA1/2, 111 lncRNAs, and six miRNAs) and 250 edges.

We first performed an integrated analysis for paired BRCA1/2 expression profiles, miRNA expression profiles and lncRNA expression profiles of 242 patients with wild-type BRCA1/2 and experimentally validated target relationship among BRCA1/2, miRNA and lncRNAs. As described in the “Materials and Methods” section, we identified a total of 83 miRNA-directed BRCA1-lncRNA ceRNA triples (including 70 lncRNAs and three miRNAs) and 66 miRNA-directed BRCA2-lncRNA ceRNA triples (49 lncRNAs and three miRNAs). Then these miRNA-directed BRCA1/2-lncRNA ceRNA triples were integrated to build a global BRCA1/2-directed ceRNA network (BRCA-CeNet). The constructed BRCA-CeNet contained 119 nodes (including BRCA1/2, 111 lncRNAs, and six miRNAs) and 250 edges (Figure 1 and Supplementary Table S1).

### Development and Validation of a BRCA1/2-Directed lncRNA Signature

We performed univariate Cox regression analysis to evaluate the association between lncRNAs in a BRCA1/2-directed ceRNA network and survival in the training dataset and identified five BRCA1/2-directed lncRNAs (*LINC01619*, *DLX6-AS1*, *AC016747.4*, *AC027290.3*, and *AC004943.2*) which are significantly associated with survival. Then we performed stepwise regression analysis for these five BRCA1/2-directed prognostic lncRNAs and identified an optimal combination of three lncRNAs (*LINC01619*, *DLX6-AS1*, and *AC004943.2*) according to AIC (Table 1). Three optimal prognostic lncRNAs were fitted in a multivariate Cox regression analysis to evaluate their relative contribution to survival prediction. Finally, a BRCA1/2-directed lncRNA signature (BRCALncSig) comprised of three lncRNAs were constructed as follows:*BRCAL*ncSig = (−0.571*EX_*LINC*01619_) + (−0.26*EX_*DLX*6−*AS*1_) + (−0.284*EX_*AC*004943.2_).

**TABLE 1:** The detailed information of three prognostic lncRNAs in the BRCA1/2-directed ceRNA network.

Ensemble ID	Gene name	Genomic location	Coefficient^a^	Hazard ratio^b^	*p*-value^b^
ENSG00000257242	LINC01619	Chr12: 91,984,976–92,142,914 (−)	−0.571	0.56	0.004
ENSG00000231764	DLX6-AS1	Chr 7: 96,955,141–97,014,088 (−)	−0.26	0.74	0.012
ENSG00000259768	AC004943.2	Chr 16: 72,665,123–72,822,781 (+)	−0.284	0.77	0.039

*^*a*^Derived from multivariate Cox regression analysis. ^*b*^Derived from univariate Cox regression analysis.*

**FIGURE 2:**
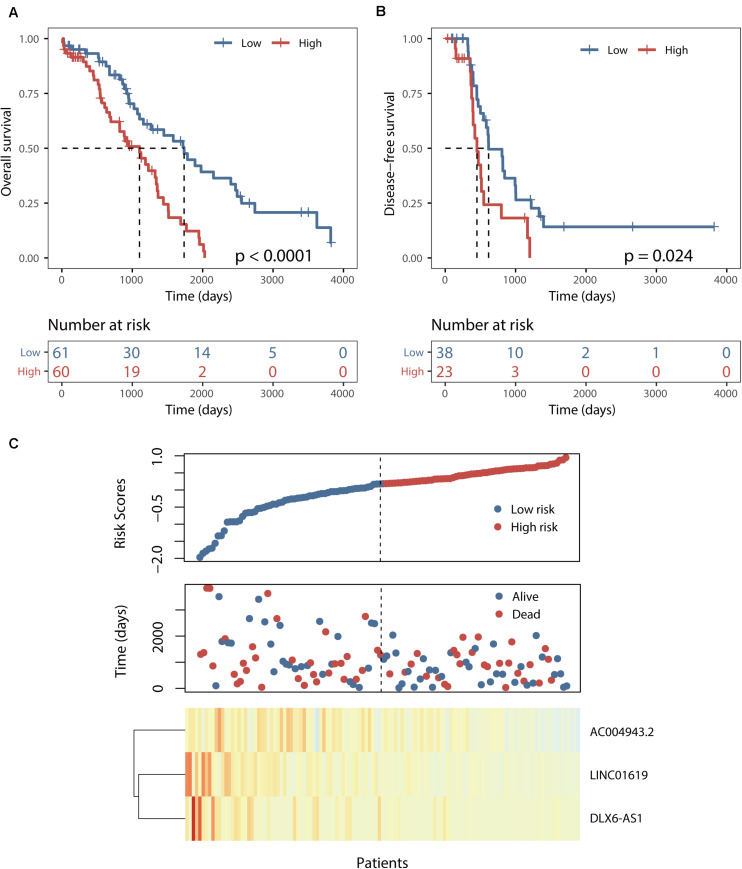
Identification of a BRCA1/2-directed lncRNA signature (BRCALncSig) in the training set. **(A)** Kaplan–Meier survival curves of overall survival between high-risk and low-risk patients. **(B)** Kaplan–Meier survival curves of disease-free survival between high-risk and low-risk patients. **(C)** Risk scores distribution and expression pattern of the BRCALncSig.

This BRCALncSig could stratify 121 patients of training dataset into two risk groups using the median value (0.175) as risk cutoff. As shown in Figure 2A, patients with high BRCALncSig tend to be high-risk, while patients with low BRCALncSig are low-risk. High-risk patients have significantly poor overall survival and disease-free survival compared with those with low-risk (log-rank *p* < 0.001 and *p* = 0.024) (Figures 2A,B). The median overall and disease-free survival time of patients in the high-risk group was 1102 days and 450 days, whereas the corresponding median survival time in the low-risk group was 1736 days and 616 days. The overall and disease-free survival rate of patients at 5 years in the high-risk group was 12% and 0%, respectively, which is significantly lower than those (45% and 14%) patients in the low-risk group. The expression pattern of BRCALncSig, scores distribution and survival time of 121 patients of training dataset were shown in Figure 2C. We found that three prognostic factors are protective factors that were significantly highly expressed in low-risk patients compared with high-risk patients (Figure 2C and Supplementary Figure S1).

### Further Validation of the BRCALncSig in the Independent Testing and TCGA Datasets

**FIGURE 3:**
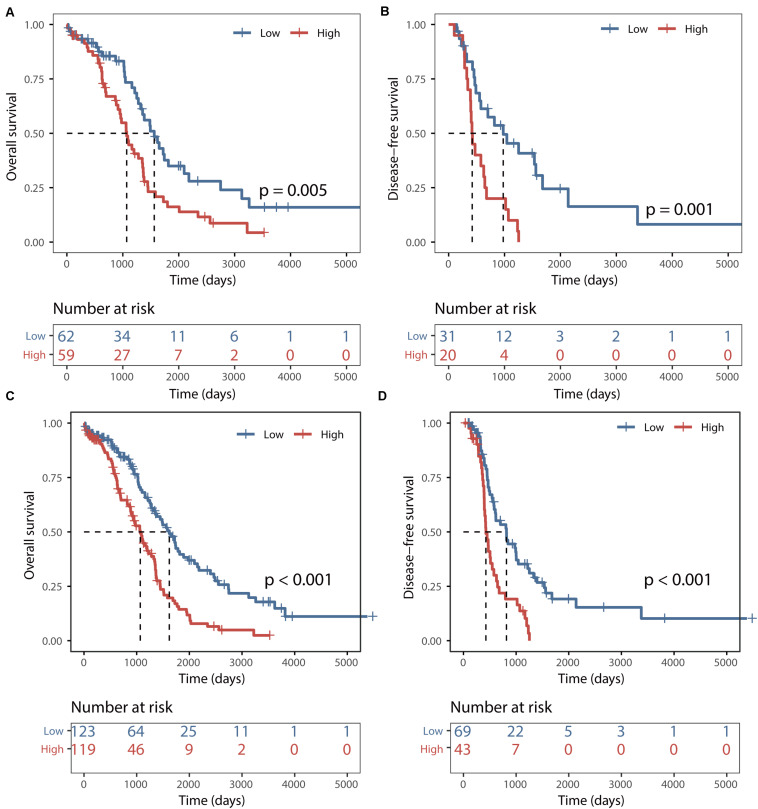
Validation of the BRCALncSig. Kaplan–Meier survival curves of overall survival between high-risk and low-risk patients in the testing set **(A)** and entire TCGA set **(C)**. Kaplan–Meier survival curves of disease-free survival between high-risk and low-risk patients in the testing set **(B)** and entire TCGA set **(D)**.

The predictive power of the BRCALncSig was further validated in another completely independent testing dataset. Using the same score model and risk cutoff derived from the training dataset without parameters re-estimation, all 121 patients of the testing dataset were divided into the high-risk group and low-risk group with significantly different overall survival (log-rank *p* = 0.005, Figure 3A) and disease-free survival (log-rank *p* = 0.001, Figure 3B). As shown in Figures 3A,B, the median overall and disease-free survival time of patients in the high-risk group was 1069 days and 422 days, which is significantly lower than that (1562 and 977 days) of patients in the low-risk group. The overall and disease-free survival rate of patients at 5 years in the high-risk group was 16% and 0%, respectively, whereas the corresponding rates are 35% and 24%, respectively. Similar results were observed for the TCGA dataset, with the median overall and disease-free survival time of patients in the high-risk group was 1069 days and 426 days, which is significantly lower than that (1620 and 817 days) of patients in the low-risk group. The overall and disease-free survival rate of patients at 5 years in the high-risk group was 14% and 0%, respectively, whereas the corresponding rates are 40% and 19% in the low-risk group, respectively (Figures 3C,D).

### Prognostic Differences Among Patients With BRCA1/2 Mutation, BRCALncSig-Related High-Risk Patients and BRCALncSig-Related Low-Risk Patients

**FIGURE 4:**
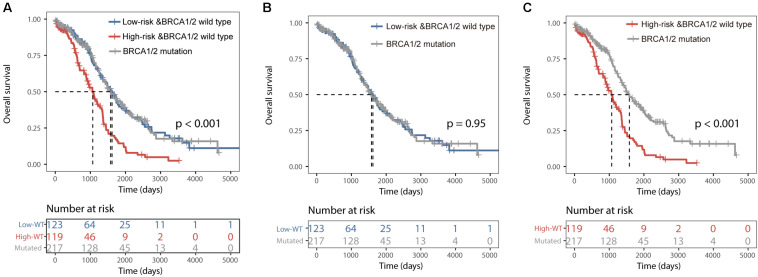
Prognostic differences among three group patients. **(A)** Kaplan–Meier survival curves of overall survival among BRCA1/2 mutation group, the BRCALncSig-related high-risk wild-type group and the BRCALncSig-related low-risk wild-type group. **(B)** Kaplan–Meier survival curves of overall survival between BRCA1/2 mutation group and BRCALncSig-related low-risk wild-type group. **(C)** Kaplan–Meier survival curves of overall survival between the BRCA1/2 mutation group and the BRCALncSig-related high-risk wild-type group.

All 459 patients of the TCGA dataset were grouped into three categories: the BRCA1/2 mutation group (*n* = 217), the BRCALncSig-related high-risk wild-type group (*n* = 119) and the BRCALncSig-related low-risk wild-type group (*n* = 123). We found that there is a significant difference in survival time among these three patient groups (log-rank *p* < 0.001) (Figure 4A). The pairwise comparison among three patient groups showed that patients with BRCA1/2 mutation and patients in the BRCALncSig-related low-risk wild-type group both have significantly longer survival than patients in the BRCALncSig-related high-risk wild-type group (log-rank *p* < 0.001) (Figure 4C). The median survival time of patients with BRCA1/2 mutation and patients in the BRCALncSig-related low-risk wild-type group is 1579 days and 1620 days, respectively, while corresponding median survival time in the BRCALncSig-related high-risk wild-type group is 1069 days. However, patients with BRCA1/2 mutation showed no significant difference in survival from patients in the BRCALncSig-related low-risk wild-type group (Log-rank *p* = 0.95, median survival time 1620 vs. 1579 days; Figure 4B).

### Correlation Between the BRCALncSig and Chemo-Response

**FIGURE 5:**
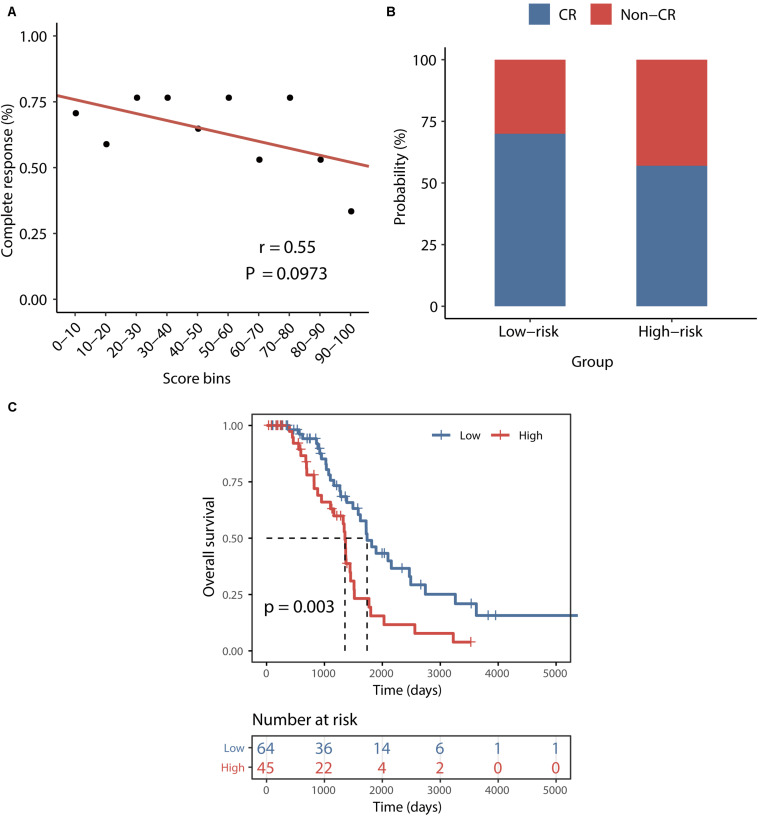
Association between the BRCALncSig and chemo-response. **(A)** The relationship between the BRCALncSig and the likelihood of complete response. **(B)** Differences in complete response ratios between the high-risk group and low-risk group. **(C)** Kaplan–Meier survival curves of overall survival between high-risk and low-risk patients for patients with CR.

To examine whether the BRCALncSig correlated with the likelihood of CR, we plotted the percentage of patients achieving CR against the BRCALncSig and found that there is a marginally significant negative correlation between the BRCALncSig and CR (Pearson correlation coefficient *r* = 0.55, *p* = 0.097) (Figure 5A). Patients in the low-risk groups tended to have a higher likelihood of CR compared with those in the high-risk groups. In detail, 70% of patients in the low-risk group achieved CR to a platinum and taxane regimen, whereas the corresponding rate is 57% in the high-risk group (*p* = 0.078, Chi-squared test) (Figure 5B). Furthermore, the BRCALncSig was also capable of discriminating nonequivalent outcomes for all patients achieving CR. As shown in Figure 5C, patients in the high-risk group have significantly shorter survival time (median 1354 days) than that of patients in the low-risk group (median 1736 days) (log-rank *p* = 0.003; Figure 5C) even if these patients all received CR to a platinum and taxane regimen.

### Independence of the BRCALncSig From Other Clinicopathological Factors

To further examine whether the BRCALncSig was independent of other clinicopathological factors, we performed multivariate Cox regression analysis for the BRCALncSig and other clinicopathological factors, including age, stage, grade and treatment response in each dataset. The results of the training set showed that the BRCALncSig (HR = 2.04, 95 CI 1.08–3.88, *p* = 0.029), age (HR = 2.17, 95% CI 1.17–4.05, *p* = 0.015), and treatment response (HR = 0.32, 95% CI 0.17–0.61, *p* < 0.001) was significantly correlated with overall survival (Table 2). Similar results also were observed in the testing dataset and entire TCGA dataset. The BRCALncSig still maintained a significant correlation with overall survival (HR = 1.95, 95% CI 1.06–3.57, *p* = 0.031 for testing dataset and HR = 1.95, 95% CI 1.27–2.98, *p* = 0.002 for entire TCGA dataset) after adjusted by age, stage, grade and treatment response in the testing and entire TCGA datasets (Table 2). The results of the multivariable Cox regression analysis thus indicated that the BRCALncSig was independent of other clinicopathological factors.

**TABLE 2:** Univariate and multivariate Cox regression analysis of overall survival in each dataset.

Variables		Univariate analysis			Multivariate analysis		
		HR	95% CI of HR	*p*-value	HR	95% CI of HR	*p*-value
Training dataset (*n* = 121)							
BRCALncSig	High/Low	2.73	1.65–4.51	<0.001	2.04	1.08–3.88	0.029
Age	>60/≤60	1.59	0.99–2.55	0.056	2.17	1.17–4.05	0.015
Stage	III,IV/I,II	2.46	0.6–10.15	0.21	2.09	0.28–15.84	0.47
Grade	G3,G4/G1,G2	1.35	0.67–2.73	0.4	1.52	0.6–3.85	0.38
Treatment response	CR/non-CR	0.34	0.19–0.6	<0.001	0.32	0.17–0.61	<0.001
Testing dataset (*n* = 121)							
BRCALncSig	High/Low	1.9	1.21–2.99	0.005	1.95	1.06–3.57	0.031
Age	>60/≤60	1.55	0.99–2.44	0.058	1.93	1.05–3.53	0.034
Stage	III,IV/I,II	2.17	0.53–8.86	0.28	1.26	0.14–10.97	0.84
Grade	G3,G4/G1,G2	1.17	0.62–2.23	0.62	1.35	0.58–3.17	0.49
Treatment response	CR/non-CR	0.32	0.18–0.57	<0.001	0.35	0.19–0.64	<0.001
TCGA dataset (*n* = 242)							
BRCALncSig	High/Low	2.17	1.56–3.01	<0.001	1.95	1.27–2.98	0.002
Age	>60/≤60	1.55	1.13–2.13	0.007	2.15	1.41–3.26	<0.001
Stage	III,IV/I,II	2.28	0.84–6.16	0.11	1.6	0.38–6.73	0.52
Grade	G3,G4/G1,G2	1.27	0.79–2.03	0.32	1.46	0.81–2.65	0.21
Treatment response	CR/non-CR	0.33	0.22–0.49	<0.001	0.32	0.21–0.5	<0.001

## Discussion

Ovarian cancer is a complex disease characterized by heterogeneous molecular and clinical features (Cancer Genome Atlas Research and Network, 2011). The clinical outcome and chemotherapy response of OvCa are very different after standard treatment because of molecular heterogeneity. The clinical variables, such as stage, grade, and age, showed significant limitations in prognosis prediction and treatment decisions of the individual patient (Zhao et al., 2020). Many clinical studies have found that BRCA1/2 mutation was associated with higher rates of response to first-line platinum-based chemotherapy and improved outcome since deficiencies in BRCA1 and/or BRCA2 usually exhibit an impaired ability in HR-mediated double-strand break (DSB) repair which are highly prone to chromosomal damage induced by platinum-based chemotherapy (Pothuri, 2013; Lu et al., 2014; Rimar et al., 2017). However, recent studies indicated that BRCA1/2 wild type patients might have HR deficiency and favorable prognosis because the expression of BRCA1/2 could be affected not only by mutation but also by other molecular mechanisms, which perturbing DNA damage response and DNA repair pathways. For example, miRNAs have been reported to target BRCA1/2 and imped DNA damage repair in OvCa cells, which could impact chemotherapeutic sensitivity (Moskwa et al., 2011; Sun et al., 2013). Recent experimental studies found that several lncRNAs also participated in the DNA damage response and DNA repair pathways by interacting with BRCA1/2. However, genome-wide screening of BRCA1/2-related lncRNAs and their clinical significance is still unexplored.

In this study, we proposed a novel computational method to identify BRCA1/2-related lncRNAs based on the “ceRNA hypothesis.” Using this method, we constructed a global BRCA1/2-directed ceRNA network by integrating paired lncRNA expression profiles, miRNA expression profiles and BRCA1/2 expression profiles in BRCA1/2 wild-type patients and identified BRCA1/2-related 111 lncRNAs. These lncRNAs may be involved in DNA damage response and DNA repair pathways by interacting with BRCA1/2 via a ceRNA mechanism. Therefore, these BRCA1/2-related lncRNAs have the potential to serve as molecular biomarkers for predicting prognosis and chemotherapy response of BRCA1/2 wild-type OvCa patients. To clarify the clinical potential of these novel BRCA1/2-related lncRNAs, we examined the association of BRCA1/2-directed lncRNAs with survival and identified five BRCA1/2-directed lncRNAs (*LINC01619*, *DLX6-AS1*, *AC016747.4*, *AC027290.3*, and *AC004943.2*) significantly correlated with survival of BRCA1/2 wild-type patients in the training dataset. Then a BRCA1/2-directed lncRNA signature (BRCALncSig) comprised of three BRCA1-directed lncRNAs (*LINC01619*, *DLX6-AS1*, and *AC004943.2*) were constructed using the stepwise regression and multivariate regression methods. The BRCALncSig revealed robust prognostic value for predicting the overall survival of patients with wild-type BRCA1/2 in training, testing and TCGA datasets, which classified the patients into two groups with significantly different overall survival. Although a relatively high ratio of OvCa patients received CRs for platinum/taxane-based chemotherapy, a portion of patients receiving CR still has a poor five-survival rate because of recurrent disease (Lu et al., 2014; Gu et al., 2015; Sun J. et al., 2019). Therefore we further examined whether this BRCALncSig was able to distinguish patients with better outcomes from those with poor outcomes after platinum/taxane-based chemotherapy. Our results demonstrated the significant association between the BRCALncSig and CR, implying the potential application of the BRCALncSig in predicting CR and platinum-resistant patients. When the BRCALncSig was applied to patients with CR, we found that the BRCALncSig was able to identify those patients who will benefit from platinum-based chemotherapy. The BRCALncSig could stratify patients with CR into cases of significantly better outcomes and cases of poor outcome. Further, there are significantly different survival differences between high-risk BRCA1/2 wild-type patients and those with BRCA1/2 mutation, and no differences in survival time between low-risk BRCA1/2 wild-type patients and those with BRCA1/2 mutation.

**FIGURE 6:**
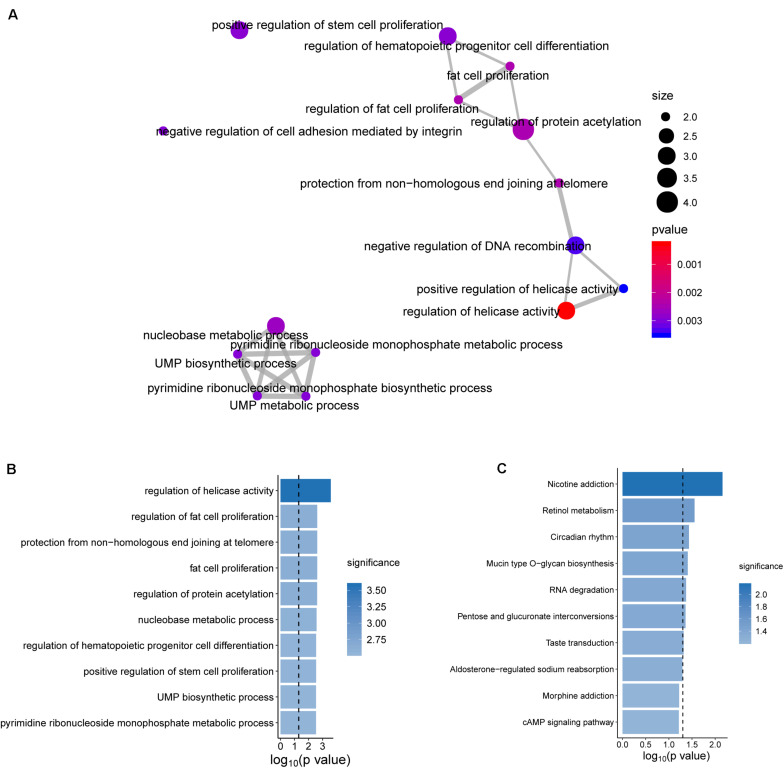
Functional enrichment analysis. **(A)** The functional map of enriched GO terms. **(B)** Enriched Go terms. **(C)** Enriched KEGG pathways.

To further explore the functional roles of the BRCALncSig in OvCa, we examined the correlation between mRNAs and the BRCALncSig, and selected the top 100 mRNAs as lncRNAs-related mRNAs. Then we performed GO and KEGG functional enrichment analysis of lncRNAs-related mRNAs, and found that these lncRNAs-related mRNAs were enriched in cancer-related or cell proliferation-related biological processes and pathways (Figure 6), provided the supporting evidence that the BRCALncSig are associated with OvCa and HR. Of three lncRNAs in the BRCALncSig, lncRNA *DLX6-AS1* has been observed to be dysregulated in several cancers (Huang et al., 2019; Sun W. et al., 2019; Zhang et al., 2019). A recent study found that lncRNA *DLX6-AS1* also plays an important role in the development and progression of OvCa (Zhao and Liu, 2019). The down-regulation of lncRNA DLX6-AS1 inhibits proliferation and metastasis via the Notch Signaling Pathway in human epithelial OvCa cells (Zhao and Liu, 2019). LncRNA *LINC01619* has been reported to be involved in the regulation of Oxidative stress (Wang et al., 2019).

In conclusion, we construed a BRCA1/2-directed lncRNA-associated ceRNA network by integrating paired lncRNA expression profiles, miRNA expression profiles and BRCA1/2 expression profiles in BRCA1/2 wild-type patients, and identified a BRCA1/2-directed lncRNA signature (BRCALncSig) that are significant with overall survival and chemotherapy response of BRCA1/2 wild-type patients. The BRCALncSig is independent prognostic factors which not only can robustly predict the survival of patients with wild-type BRCA1/2, but also identify OvCa patients who will benefit from platinum-based chemotherapy. However, further validation and investigation into the molecular mechanisms of how lncRNAs interact with BRCA1/2 to regulate HR and their impact on the prognosis and chemo-response should be done for in prospective studies.

## Data Availability Statement

Publicly available datasets were analyzed in this study. This data can be found here: Clinical information, miRNA expression profiles, lncRNA expression profiles, mRNA expression profiles, somatic mutation, and copy number variation information of BRCA1 and BRCA2 of ovarian cancer patients were obtained from UCSC Xena (https://xena.ucsc.edu/).

## Author Contributions

DW conceived and designed the experiments and wrote the manuscript. MZ, GW, and YZ analyzed the data. All authors read and approved the final manuscript.

## Conflict of Interest

The authors declare that the research was conducted in the absence of any commercial or financial relationships that could be construed as a potential conflict of interest.

## Footnotes

1 https://xena.ucsc.edu/

2 http://www.microrna.gr/tarbase

3 http://miRTarBase.cuhk.edu.cn/

4 http://starbase.sysu.edu.cn/

## Supplementary Material

FIGURE S1 Expression pattern of three lncRNA biomarkers between low-risk patients and high-risk patients.

TABLE S1 Global BRCA1/2-directed ceRNA network.
