# Supplementary figures and images for: Characterization of BRCA1/2-Directed ceRNA Network Identifies a Novel Three-lncRNA Signature to Predict Prognosis and Chemo-Response in Ovarian Cancer Patients With Wild-Type BRCA1/2
Source: Front Cell Dev Biol. 2020 Jul 29;8:680. doi: 10.3389/fcell.2020.00680 (PMC7403448; doi:10.3389/fcell.2020.00680)

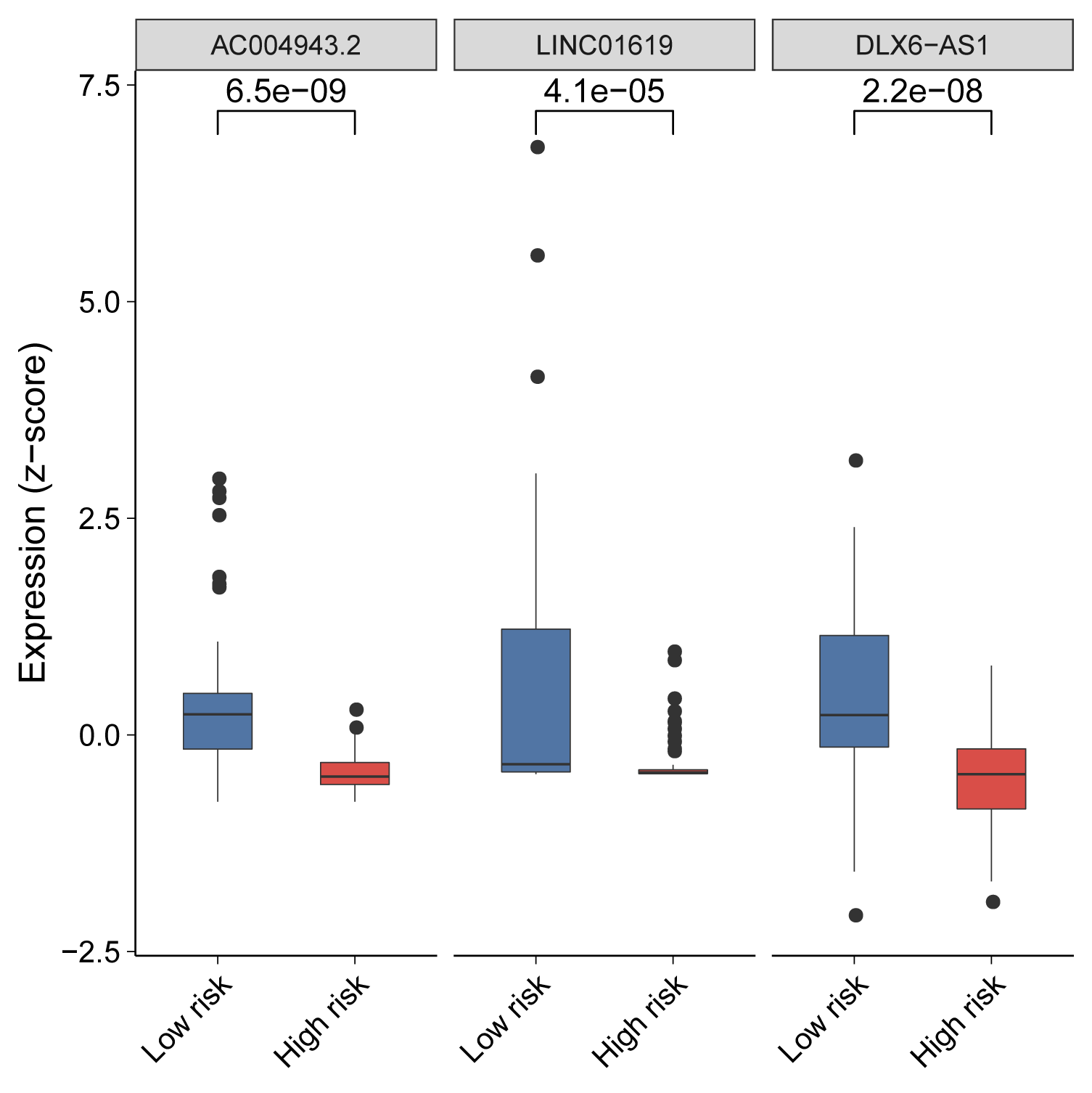

Supplement: FIGURE S1 — Expression pattern of three lncRNA biomarkers between low-risk patients and high-risk patients. [file Image_1.TIF]
